# Uveal Melanoma Cells Utilize a Novel Route for Transendothelial Migration

**DOI:** 10.1371/journal.pone.0115472

**Published:** 2014-12-15

**Authors:** Michael D. Onken, Jinmei Li, John A. Cooper

**Affiliations:** Department of Cell Biology & Physiology, Washington University School of Medicine, St. Louis, Missouri, United States of America; University of Tennessee, United States of America

## Abstract

Uveal melanoma arises in the eye, and it spreads to distant organs in almost half of patients, leading to a fatal outcome. To metastasize, uveal melanoma cells must transmigrate into and out of the microvasculature, crossing the monolayer of endothelial cells that separates the vessel lumen from surrounding tissues. We investigated how human uveal melanoma cells cross the endothelial cell monolayer, using a cultured cell system with primary human endothelial cell monolayers on hydrogel substrates. We found that uveal melanoma cells transmigrate by a novel and unexpected mechanism. Uveal melanoma cells intercalate into the endothelial cell monolayer and flatten out, assuming a shape and geometry similar to those of endothelial cells in the monolayer. After an extended period of time in the intercalated state, the uveal melanoma cells round up and migrate underneath the monolayer. VCAM is present on endothelial cells, and anti-VCAM antibodies slowed the process of intercalation. Depletion of BAP1, a known suppressor of metastasis in patients, increased the amount of transmigration of uveal melanoma cells in transwell assays; but BAP1 depletion did not affect the rate of intercalation, based on movies of living cells. Our results reveal a novel route of transendothelial migration for uveal melanoma cells, and they provide insight into the mechanism by which loss of BAP1 promotes metastasis.

## Introduction

Melanomas are highly aggressive cancers that often metastasize and result in patient death [1]. For melanomas that arise in the pigmented uveal layers of the eye, almost half of patients develop fatal metastatic disease, even after the tumor-bearing eye is surgically removed [2]. Uveal melanomas (UMs) present as discrete masses between the thick, fibrous sclera and the retina, often pushing the retina into the vitreous space [3]. The highly vascular nature of the uvea provides a ready outlet for the spread of UM cells to distant organs through the bloodstream [4]. The anatomy of the eye, including its notable lack of lymphatic vessels, implies that local spread of UM to surrounding tissues is rare [5]. Therefore, the spread of UM cancer cells by the hematogenous route is critical to the morbidity and mortality of the disease, and it would be important to understanding the mechanism by which UM cells cross the endothelial barrier as they enter and exit the bloodstream.

Despite advances in treatment for the primary tumor in the eye, the mortality rate for UM has not changed, due to our inability to prevent or treat metastases [6]. UM cells have been detected in the circulation of patients at the time of diagnosis and after removal of the primary tumor. Patients with clinically detectable metastatic disease also have circulating UM cells [7], and the number of circulating cells correlates with the size and number of metastatic lesions [8]. Surprisingly, circulating UM cells have also been found at the time of diagnosis of the primary tumor in patients who did not proceed to develop metastases [7], [9]. Therefore, the ability of UM cells to enter the bloodstream appears not to predict the ability of the cells to metastasize, suggesting that the metastatic potential of a given tumor depends on the ability of the cells to exit the bloodstream via the microvasculature, in order to invade and colonize distant organs.

The vessels of the microvasculature are important conduits for exchange of metabolites, and they provide immune cells with access to tissues. The endothelial cells (ECs) that line these vessels inform circulating immune cells of the state of the surrounding tissue, and they recruit and activate immune cells to migrate into the surrounding tissue under a variety of physiological and pathological conditions [10]. ECs maintain the integrity of the vasculature during transendothelial migration (TEM) by immune cells by creating, maintaining and closing passages for immune cells to cross the EC monolayer [11]–[13].

Cancer cells appear to co-opt the process of TEM when they transit the vessel wall, entering and leaving the circulation [14]–[16]. These transmigration events can occur in the absence of inflammatory signals, suggesting that other factors, such as mechanical stress or properties of the surrounding tissue, may help to regulate the timing and efficiency [17]. We have developed a cell-culture model of TEM using monolayers of primary human dermal microvascular ECs (HDMVECs) on hydrogels with stiffnesses similar to normal human tissue [18]. Here, we used this model to study the transmigration of UM cells across EC monolayers. We report the discovery that UM cells transmigrate via a novel process that includes intercalation into the EC monolayer, after which they migrate under the monolayer to invade interstitial tissue. We find that this process requires VCAM-mediated adhesion between UM cells and ECs and that loss of the metastasis suppressor BAP1 enhances TEM.

## Materials and Methods

Chemicals and reagents were obtained from Fisher Scientific (Pittsburgh, PA) or Sigma-Aldrich (Saint Louis, MO), unless stated otherwise.

### Cell Culture

Cells were cultured at 37°C in 5% CO_2_. Primary HDMVECs were obtained from Lonza (Allendale, NJ) and cultured in EBM-2 base medium supplemented with the EGM-2 MV kit. HDMVECs were not used after 9 passages. Human 92.1 and OCM-1A UM cells were derived by and the generous gifts of Drs. Martine Jager (Laboratory of Ophthalmology, Leiden University) [19] and June Kan-Mitchell (Biological Sciences, University of Texas at El Paso) [20], respectively. Both cell lines were grown in RPMI 1640 medium (Life Technologies, Carlsbad, CA) supplemented with 10% FBS and antibiotics.

### Lentiviral Constructs and Plasmids

Lentiviral-based constructs were used for expression and knockdown experiments. Lentiviral pBOB-GFP was used to express GFP in HDMVEC cells. Lentiviral pLKO.1 shRNA expression plasmids targeting GFP (clonetechGfp-438s1c1) and BAP1 (NM_004656.2-2658s1c1) were obtained from The RNAi Consortium (TRC) via the Children's Discovery Institute/Genome Sequencing Center at Washington University. Viral production and infections were carried out according to consortium recommendations (Broad Institute). The F-tractin expression construct (ptdTomato-N1-F-tractin) [21] was a generous gift of Dr. Michael Schell (Department of Neurology, Uniformed Services University of the Health Sciences).

### Immunostaining and antibodies

Fixation was carried out by adding an equal volume of 2× fixative (PBS with 4% paraformaldehyde and 0.4% glutaraldehyde) to EC monolayers in EGM-2 MV medium 15 min, 1 hr, 3 hrs, and 6 hrs after addition of UM cells. After 15 min at 37°C, cells were permeabilized with 0.1% Triton X-100 in PBS for 5 min, washed with PBS, and blocked with 2% fish gelatin (Sigma-Aldrich) in PBS. Primary and secondary antibodies were diluted in 2% fish gelatin in PBS. Primary antibodies included mouse monoclonal anti-cortactin (Millipore, Billerica, MA), mouse monoclonal anti-E-cadherin (BD Biosciences, Franklin Lakes, NJ), mouse monoclonal anti-N-cadherin (BD Biosciences), mouse monoclonal anti-VCAM (R&D Systems, Minneapolis, MN), rabbit polyclonal anti-S100 (DakoCytomation, Denmark), and rabbit polyclonal anti-VE-cadherin (Cell Signaling, Danvers, MA). F-actin was visualized using Alexa-fluor-conjugated phalloidin (Life Technologies). Secondary antibodies were Alexa-fluor conjugates (Life Technologies), and the mounting agent was ProLong Gold (Life Technologies). Cells were imaged with an inverted microscope (Olympus IX72) using a 40× objective.

### Live Cell Imaging and Analysis

Hydrogels on coverslips were prepared with polyacrylamide as described [18]. The hydrogels were coated with fibronectin (10 µg/mL in PBS) by overnight incubation at 4°C and washed with PBS. HDMVECs were plated on the fibronectin-coated hydrogel substrates and incubated overnight to allow formation of monolayers. Monolayers were inspected by phase-contrast microscopy to ensure that ECs covered the substrate completely, without defects, before transmigration assays were performed. For blocking experiments, mouse monoclonal antibodies against ICAM1 (BBIG-I1, R&D Systems) or VCAM (BBIG-V1, R&D Systems) were diluted into EGM-2 MV media and added to monolayers 1 hr before assaying TEM.

Melanoma cells were added to HDMVEC monolayers in EGM-2 MV culture medium and placed into an environmental chamber (Stage Top Incubator, Tokai Hit, Shizuoka-ken, Japan) with 5% CO_2_ at 37°C on either an inverted wide-field microscope (Olympus IX72) or an inverted laser-scanning confocal microscope (Olympus FV1200). Wide-field fluorescence and DIC images were captured at 12-s intervals with a 10× or a 40× objective. Confocal fluorescence and DIC images were captured at 20-s intervals with a 60× objective.

### Transwell assays

Transwell chambers (Corning) with 5-µm pore-size inserts were coated with 10 µg/mL fibronectin in PBS overnight at 4°C. HDMVECs were plated on the chamber membrane by adding 0.6×10^5^ cells in 100 µL HDMVEC media. After 24 hrs, the media was replaced with fresh HDMVEC media (100 µL in the top chamber and 600 µL in the bottom chamber). For blocking experiments, antibodies against ICAM1 or VCAM as noted above were diluted into EGM-2 MV media and added to monolayers 1 hr before TEM. UM cells (10^5^ in 100 µL serum-free RPMI medium) were added to the EC monolayer. The medium in the bottom chamber was replaced with RPMI medium containing 10% FBS to act as a chemoattractant. The chambers were incubated at 37°C for 7 hrs to allow for transmigration. UM cells were collected from the bottom chamber every hr.

## Results

### UM cells Intercalate into EC Monolayers

**Figure 1:**
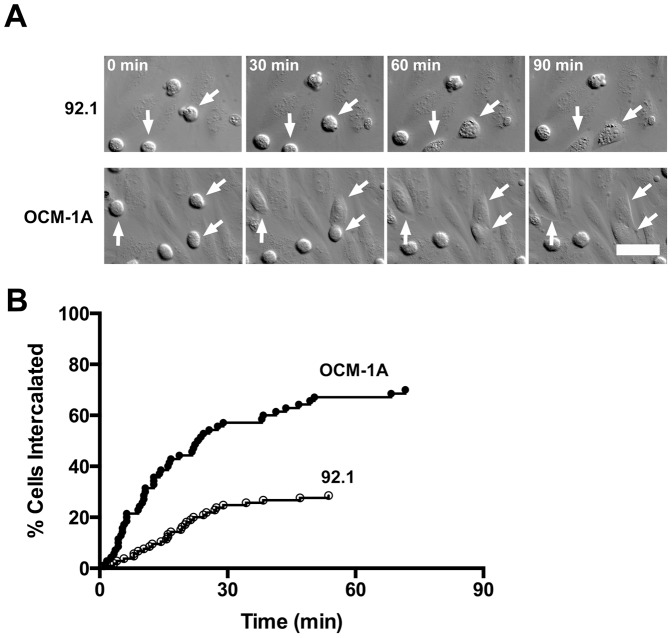
UM cells transmigrate through a novel intercalation process. A) Frames from DIC movies of UM cells, 92.1 and OCM-1A, intercalating into the endothelial monolayer (S1 Movie). Arrows indicate intercalation events. Scale bar  = 50 µm. B) Plot of the percentage of cells that achieve intercalation over time, based on the movies.

We asked how UM cells interact with monolayers of primary human ECs grown on soft substrates. Immune cells move rapidly and efficiently through EC monolayers, based on movies of living cells in several studies [12], which we confirmed in our system [22]. The behavior of migrating melanoma cells was strikingly different. UM cells, both the 92.1 and OCM-1A cell lines, inserted themselves between ECs in HDMVEC monolayers and assumed a flattened morphology, appearing to join the monolayer in the manner of an EC. (Fig. 1A, S1 Movie). This intercalation occurred exclusively at EC cell-cell junctions. No cells followed a transcellular route through a single EC, in striking contrast to the behavior of migrating immune cells [23].

The timing of the initiation of intercalation differed somewhat for the two UM cells lines (Fig. 1B). OCM-1A cells were faster, with 67% initiating TEM by 1 hr after addition, compared with 29% of 92.1 cells (Fig. 1B). Within this time frame, essentially all UM cells that intercalated into the monolayer remained in the intercalated state, without migrating through and under the monolayer or reversing course to move up and back out.

### Stepwise Progression of Intercalation

**Figure 2:**
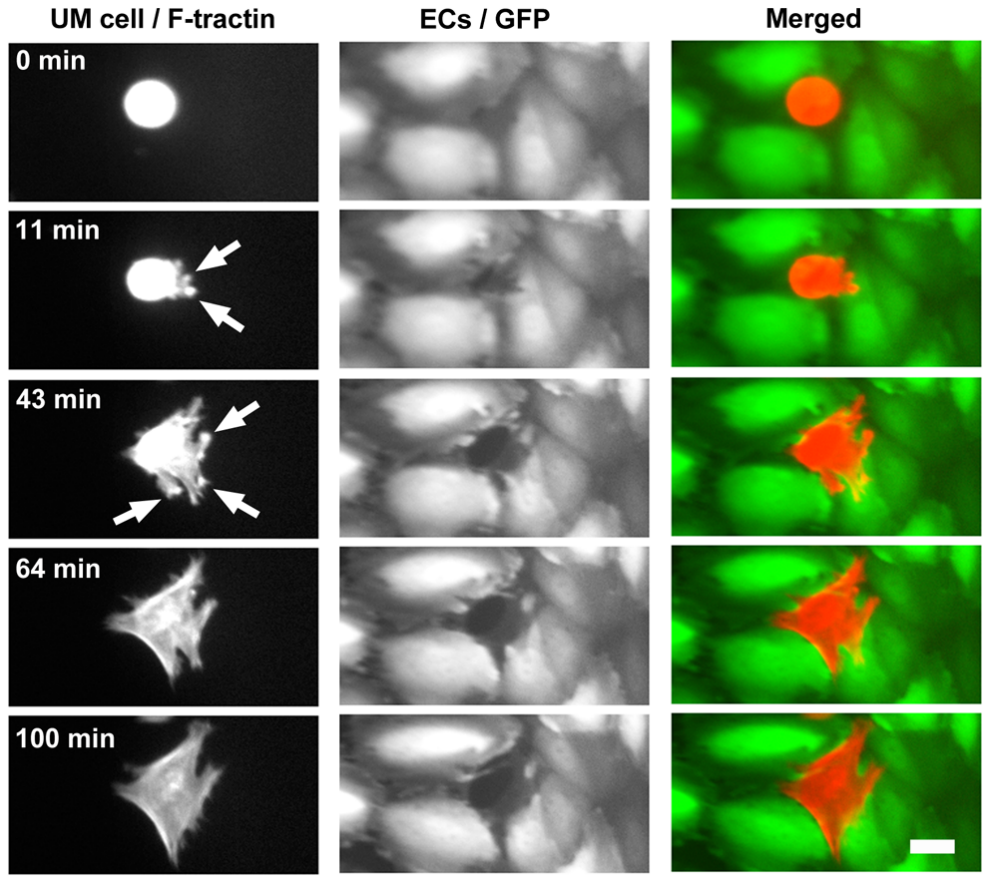
Invasive projections are rich in F-actin. Frames from S2 Movie show a UM cell (OCM-1A) with F-tractin-labeled actin filaments intercalating between ECs in a monolayer. Arrows indicate F-actin-rich protrusions that transiently probe the space under the monolayer. The UM cell completes intercalation and maintains contact with adjacent ECs. Scale bar  = 20 µm.

We examined the steps by which UM cells intercalated into EC monolayers, using dual-color live-cell imaging. We followed actin dynamics in UM cells by expressing the fluorescent marker tdTomato-F-tractin; the ECs expressed GFP. S2 Movie is a representative example, and selected frames are shown in Fig. 2. UM cells settled onto the HDMVEC monolayers and remained relatively stationary, displaying little migration across the surface of the monolayer, in contrast to the active migration displayed by immune cells [12], [22]–[24]. During this time, UM cells extended protrusions that appeared to probe the surface of the EC monolayer (Fig. 2, second row). When a UM cell protrusion contacted an EC cell-cell junction, the protrusion sometimes became attached, after which the UM cell moved to center itself over the point of contact.

Next, a small opening appeared in the EC cell junction, and the UM cell extended protrusions through the opening, which contacted the substrate (S2 Movie). The UM cell maintained contact with the substrate, and the opening between the ECs expanded (Fig. 2, third row). Eventually, the opening expanded to the size and dimensions of the UM cell, and the UM cell expanded and flattened, assuming a morphology similar to that of a typical EC in the monolayer. The UM cell maintained close contact with its EC monolayer neighbors (Fig. 2, fourth row). The UM cell remained intercalated within the monolayer for an extended period of time (Fig. 2, last row).

### UM Cell Migration after Intercalation

**Figure 3:**
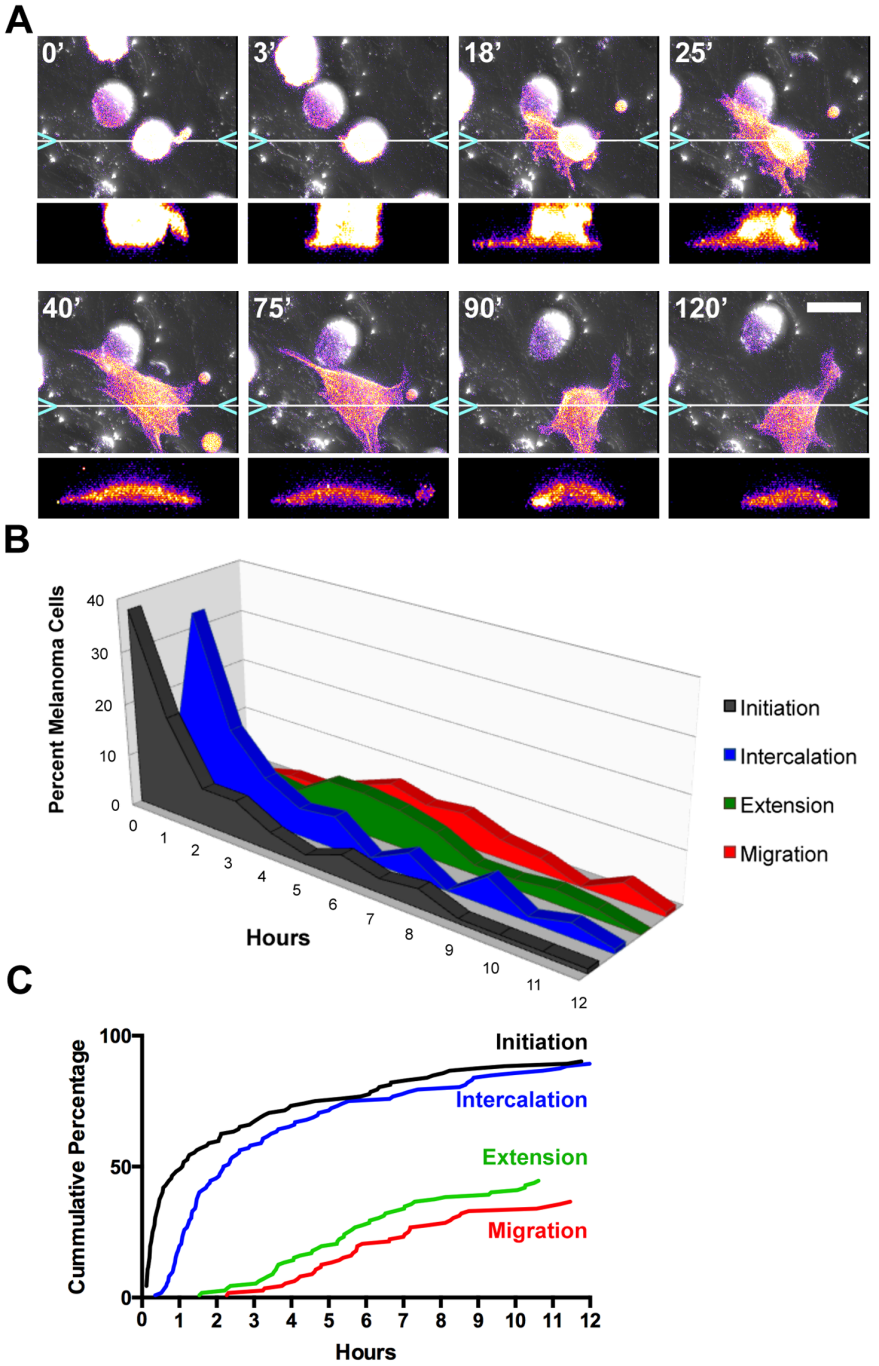
Melanoma transmigration is a stepwise process. A) Frames from S3 Movie showing transmigration of a UM cell (OCM-1A) expressing F-tractin (pseudocolored to show fluorescence intensity). Time in min is indicated in the upper left of each panel. Below each panel is an x-z slice through the intercalating cell, taken at the position indicated in the x-y image. B and C) Timing of steps of UM cell transmigration, based on results from three 12-hr movies. Panel B shows the percentage of cells in each state at a given time, and panel C plots the results as a cumulative percentage of cells that have undergone a step. Scale bar  = 25 µm.

We extended the time frame of our experiments to observe whether and how UM cells would exit the EC monolayer. During the process of intercalation, UM cells extended numerous transient projections underneath the EC monolayer (Fig. 3A, S3 Movie). As time progressed, cells in the intercalated state continued to extend these processes, released their contacts with their EC neighbors and migrated underneath the EC monolayer.

To examine the dynamics of intercalation and migration, we collected longer (12-hr) movies of UM cells on EC monolayers. We used OCM-1A cells because their rate of intercalation was higher than that of 92.1 cells. The OCM-1A cells interacted with the EC monolayers immediately on addition, as described above, with approximately half of cells initiating intercalation by 65 min (Fig. 3B). The first cells completed intercalation at 20 min after initiation, approximately half of cells intercalated by 130 min, and 90% by 12 hrs (Fig. 3B).

UM cells remained intercalated for a period of time and then migrated underneath the EC monolayer. Some UM cells remained intercalated for the duration of the movie (>11 hrs). Cells continued to extend transient protrusions under the monolayer during intercalation, but the first occurrences of persisting projections were at ∼90 min. By 12 hr, at the end of the movie, ∼45% of cells displayed persisting projections under the monolayer (Fig. 3B). UM cells began to migrate under the monolayer and away from their intercalated positions at ∼135 min, and 37% of UM cells had migrated away from their positions by 12 hr (Fig. 3B).

No UM cells moved back up and out of the monolayer, presumably because their adhesion to the fibronectin in the hydrogel substrate favored their movement under the monolayer.

### UM Transmigration is Regulated by Cell Adhesion Molecules

We asked whether UM cells initiated and maintained contact with ECs through known cell adhesion receptors. When immune cells perform TEM, they bind to ICAM1 on the surface of activated ECs [13]. For UM cell TEM, ECs are not expected to be activated in the metastatic tissue target; therefore, the EC monolayers were not treated with inflammatory activators in our system. However, ICAM1 and VCAM are expressed constitutively on ECs, albeit at lower levels than on activated ECs. In addition, ECs use VE-cadherin to adhere to each other at cell junctions. UM cells express E-cadherin and N-cadherin [25], and VE-cadherin has been observed on cutaneous melanoma cells [26].

**Figure 4:**
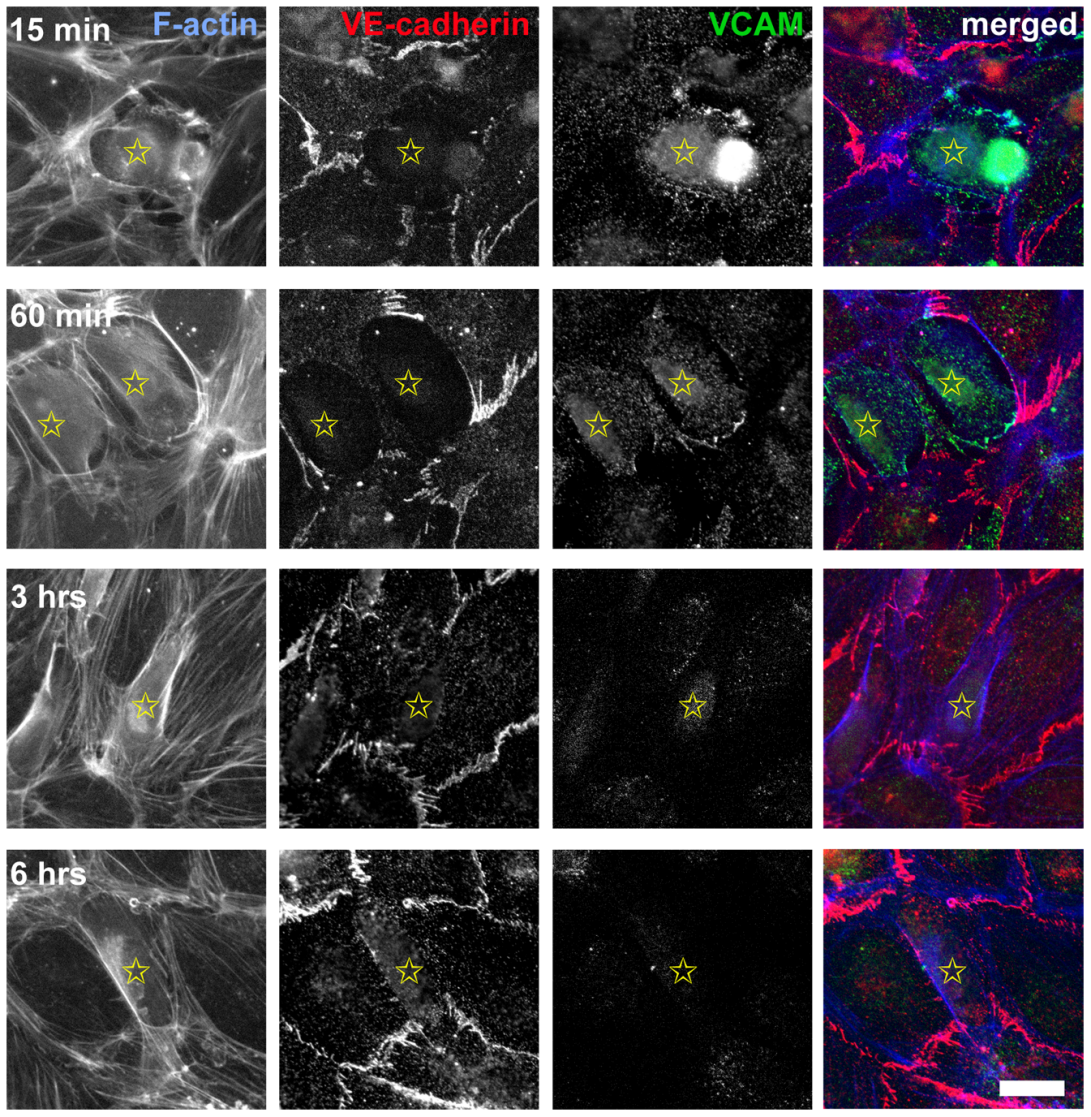
Localization of VCAM and VE-cadherin in UM cells interacting with ECs. Cell preparations were fixed and stained at indicated times after addition of UM cells. Anti-VE-cadherin stains junctions between ECs in the monolayer but not junctions between UMs and ECs. Anti-VCAM fluorescence appears transiently on ECs at the point of contact with UM cells, indicated by yellow stars, at the start of transmigration. Scale bar  = 25 µm.

We observed accumulation of VCAM on ECs at the point of their initial contact with transmigrating UM cells, by immunofluorescence (Fig. 4). This accumulation was transient, and VCAM was not observed at junctions between UM cells and ECs after intercalation was complete (Fig.ure 4). Neither VE-cadherin, E-cadherin, nor N-cadherin was observed at UM - EC junctions (Fig.ure 4 and data not shown).

We tested for functional roles by adding blocking antibodies to ICAM1 and VCAM. Anti-ICAM1 decreased the rate of intercalation by ∼50%, based on 1-hr movies (Fig.ure 5A), but the Abs had no effect on the level of TEM in 5-hr transwell assays (Fig.ure 5B). Anti-VCAM had a similar inhibitory effect in 1-hr movies; however, the level of TEM in transwell assays increased by a factor of four (Fig.ure 5). Together, these results indicate that integrin-mediated interactions are important and that intercalation into and migration out of the EC monolayer depend on different adhesive molecules.

**Figure 5:**
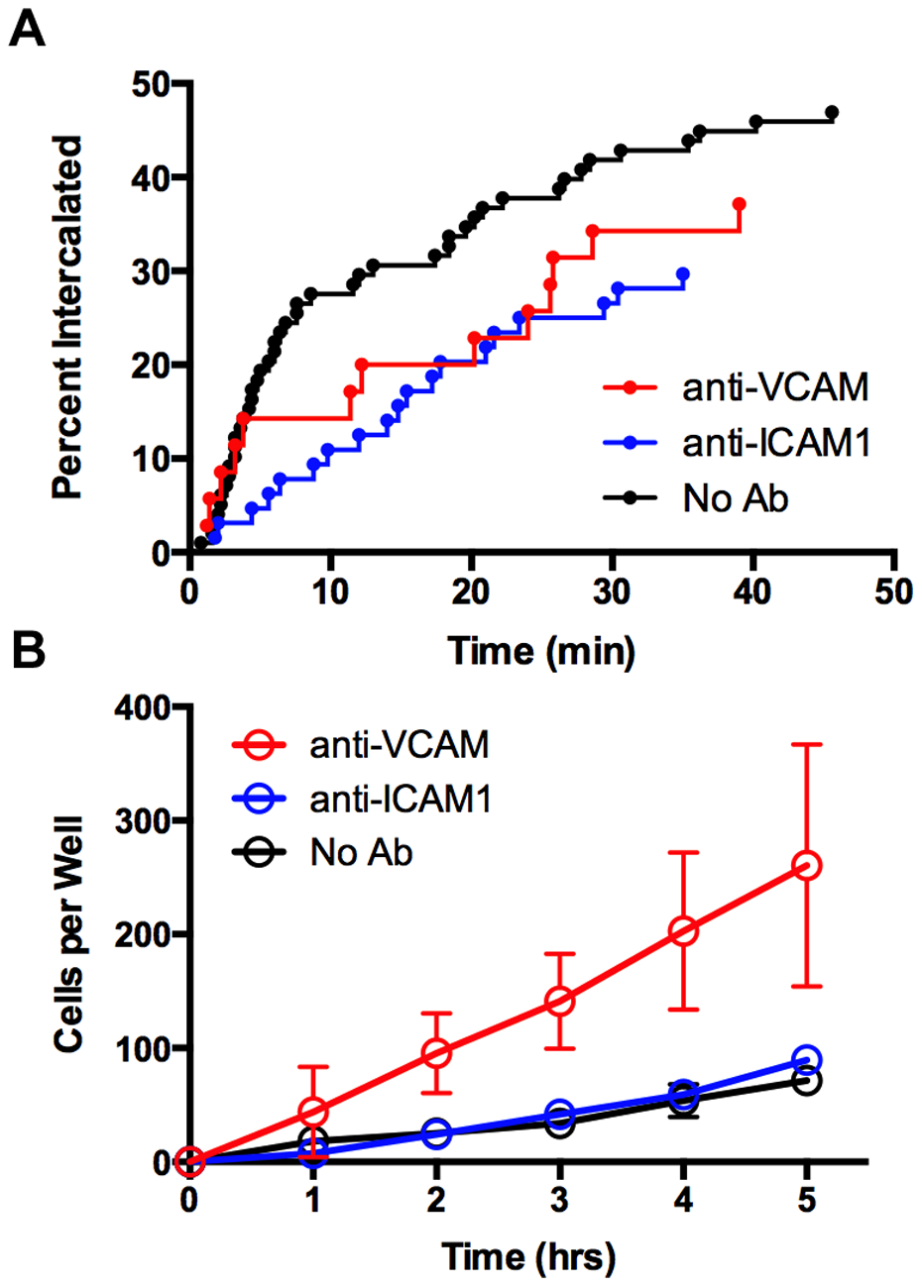
Blocking Abs against VCAM and ICAM1 affect the timing of TEM. A) Intercalation of UM cells undergoing TEM based on movies, as in Fig. 1. Blocking antibodies to ICAM1 and VCAM decreased the rate of intercalation. B) Effect of blocking Abs on bulk transmigration of UM cells assayed in transwell chambers.

### Loss of BAP1 enhances UM transmigration

*BAP1* loss-of-function mutations promote the metastatic spread of UM cancers in patients [27], and TEM should be a rate-limiting step of metastasis. Therefore, we asked whether loss of BAP1 affected transmigration of UM cells in our model of TEM. We depleted BAP1 in UM cells by lentiviral infection of cell with shRNA targeting BAP1.

First, we used conventional transwell assays, with ECs forming monolayers on the surface of the transwell filter. Depletion of BAP1 doubled the number of transmigration events by UM cells in these assays (Fig.ure 6A). Next, we used movie analysis of living cells to examine BAP1-depleted UM cells interacting with EC monolayers. The rate and efficiency of intercalation were similar to those of control UM cells over one hr (Fig.ure 6B). We conclude that the effect of loss of BAP1, seen in the transwell assay, is on later steps in transmigration, after those observed in the movie analysis of intercalation.

**Figure 6:**
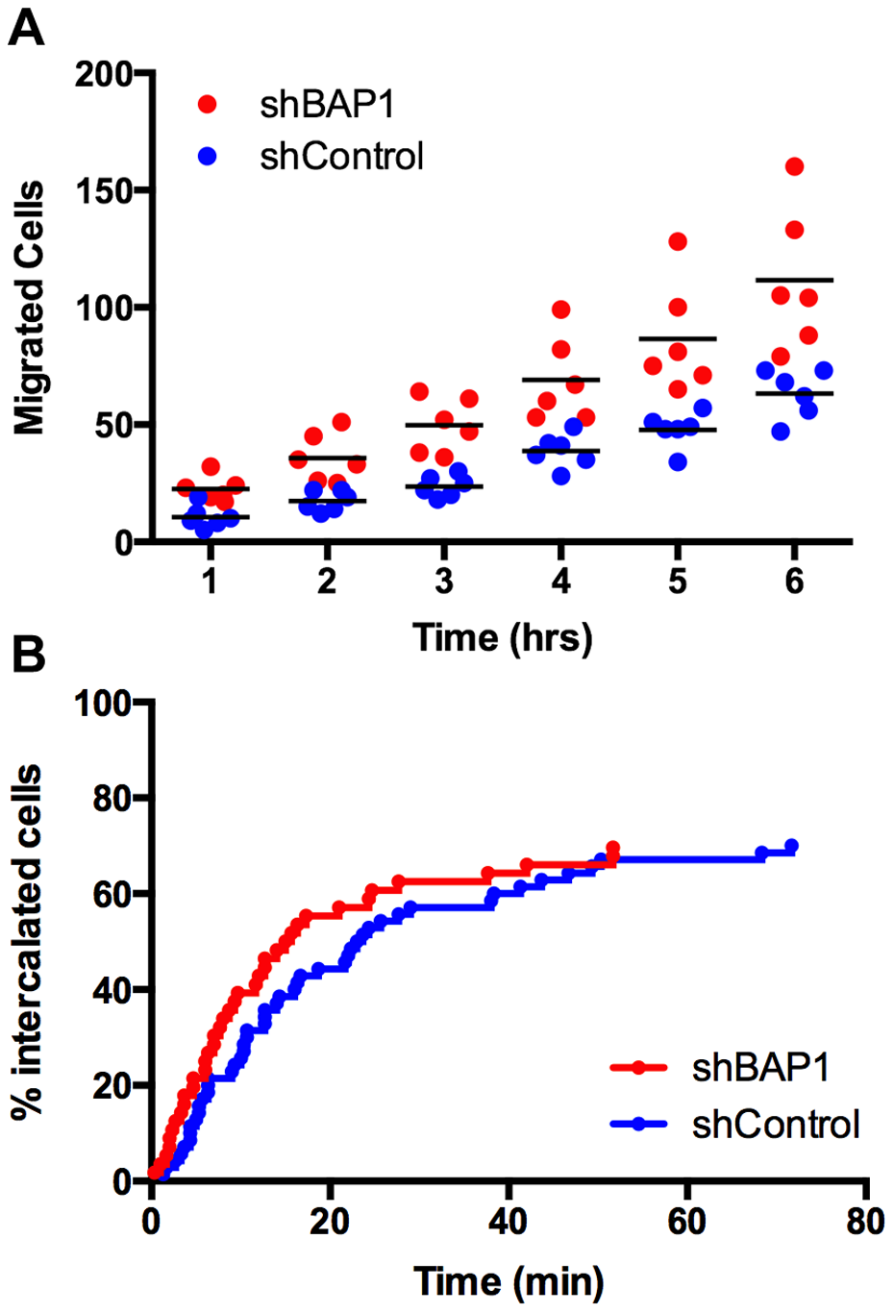
Effects of depletion of BAP1 on transendothelial migration. A) TEM of UM cells based on transwell assays after lentiviral induction of shRNA targeting BAP1 or GFP. B) Intercalation of UM cells into EC monolayers, based on movies.

## Discussion

We investigated the cellular and molecular mechanisms used by UM cells to transmigrate through EC monolayers. We made three important discoveries. First, UM cells move through the EC monolayer in a remarkable process not previously observed in studies of transendothelial migration. The UM cells intercalate into the EC monolayer, where they assume a flattened and spread morphology similar to an EC in the monolayer. They remain intercalated for an extended period time, before migrating through and underneath the monolayer. Second, we discovered a functional role for integrin-based adhesion in the intercalation process. Third, BAP1, a critical suppressor of metastasis by UM cells, has a role in the overall efficiency of transmigration, but not on the intercalation process.

### UM Cells Transmigrate Through a Series of Distinct Steps

We found that UM cells perform TEM by a specific multi-step route (diagrammed in Fig.ure 7A), which differs substantially from that taken by immune cells. First, UM cells probe the apical surface of the EC monolayer, and they extend processes that attach to cell junctions between adjacent ECs. Next, UM cells enter and enlarge the space between ECs. The UM cells proceed to flatten, while maintaining contact with the ECs, essentially intercalating themselves into the monolayer with the position and shape of an EC. The UM cells stay intercalated in the EC monolayer for a period of time. They extend processes underneath the EC monolayer, contacting the fibronectin-coated hydrogel substrate. Finally, the UM cells release their attachments to the ECs, and then move under the EC monolayer and migrate between the monolayer and the substrate.

**Figure 7:**
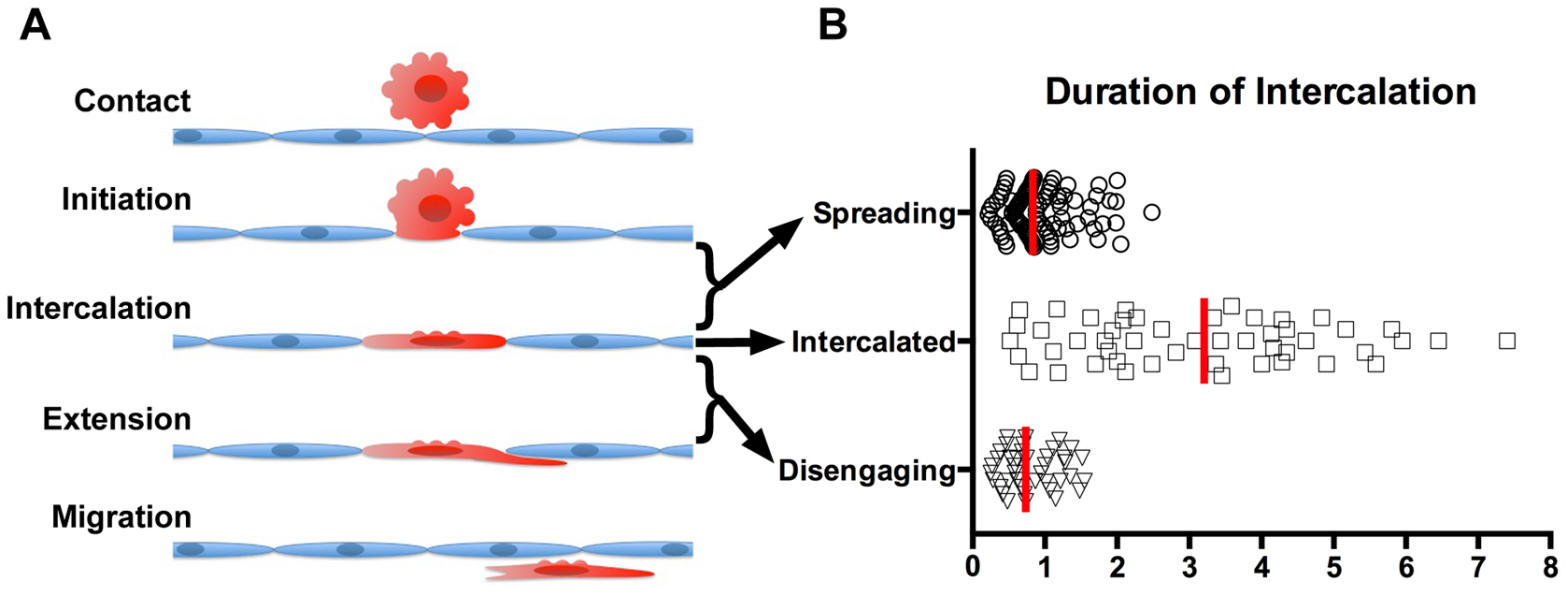
Timing of TEM steps for UM cells. A) Diagram depicting the steps of TEM used by UM cells. B) Plot of time spent in each phase of intercalation. The data include only cells that eventually disengaged from intercalation; many cells were still intercalated at 12 hrs and could not be assessed for duration of intercalation. Red bars indicate medians.

One notable feature of this process is the amount of time that the UM cells spend in the intercalated state, relative to the time used for entering and exiting the intercalated state (Fig.ure 7B). Both uveal and cutaneous melanoma cells have been observed to undergo “vasculogenic mimicry,” taking on an endothelial morphology and forming tubular networks in 3D-culture systems [28], [29]. In addition, cutaneous human melanoma cells can contribute to vascular channels in mouse xenograft tissues [30]. Cutaneous melanoma cells express VE-cadherin in the process of vasculogenic mimicry [26], but we did not observe VE-cadherin on UM cells intercalated into EC monolayers (Fig.ure 4). Thus, the intercalated state observed in our system appears to be a novel interaction between UMs and ECs distinct from vasculogenic mimicry. We speculate that the intercalation process may be a critical component of metastasis in tissues and the organism.

### Functional Roles for Integrin-based Adhesion in UM Intercalation

When immune cells cross the endothelium, immune-cell integrins bind to ICAM1 on the surface of the EC [13]. While UM cells do not express the beta2 integrins characteristic of immune cells [31], they do express several beta1 integrins [32], including alpha4beta1, which binds VCAM and is upregulated on invasive UM cells [33]. Neither UM cells nor ECs express ligands for ICAM1, so we were surprised to find an effect on intercalation with anti-ICAM1 antibodies. Antibody crosslinking of ICAM1 can activate the ICAM1 pathway in ECs [34], and this may be affecting EC activity in our movies. We observed accumulation of VCAM on ECs at the initiation of TEM, which was lost when intercalation was compete. Using blocking antibodies against VCAM and ICAM1, we observed a decreased rate of intercalation of UM cells into the EC monolayer, based on movie assays. However, anti-VCAM increased the overall level of transmigration in transwell assays, while anti-ICAM1 had no effect in this assay. VCAM and ICAM1 both contribute to the process of TEM, but to different extents and probably in different roles.

### BAP1 Depletion Enhances TEM

Human uveal melanoma tumors with loss-of-function mutations in *BAP1* are more likely to metastasize [27]. Circulating tumor cells must presumably execute TEM as part of entering tissues during the process of metastasis. Therefore, we hypothesized that decreasing BAP1 might increase the ability of UM cells to perform TEM in our model system. As predicted, shRNA-mediated depletion of BAP1 increased the overall rate of TEM by UM cells in transwell assays. However, movies of UM cells undergoing transmigration revealed no effect of BAP1 depletion on the rate of UM cell intercalation into the monolayer. Thus, BAP1 depletion appears to affect later steps in transmigration of UM cells, such as disengagement from and invasion underneath the monolayer.

The differences in rates of intercalation between the two UM cell lines were notable (Fig.ure 2B). Both the 92.1 and OCM-1A lines were derived from spindle type B tumors [19], [20], and both lines maintain this spindle morphology in culture, suggesting that the intercalation rate differences do not result from alterations in cell morphology. Depletion of BAP1 from these cell lines makes them less spindle-like in culture [35]. We show here that BAP1-depleted cells are also more invasive, suggesting a possible link between the later steps in transmigration with cell morphology.

In cutaneous melanoma, pigmentation has been associated with tumor cell function [36], poor prognosis [37], and resistance to therapies [38], [39]. In uveal melanoma, tumor pigmentation has been associated with tumor size [5], which is associated with poor prognosis [1]. The 92.1 and OCM-1A cell lines both produce melanin [19], [20], and depletion of BAP1 decreases the expression of genes involved in melanogenesis [35]. Thus, the cell survival advantage associated with increased melanin and the metastatic advantage of increased transendothelial migration appear to be independent.

### Concluding Remarks

We discovered that UM cells transmigrate through EC monolayers by a novel mechanism in which they intercalate into the EC monolayer and assume an EC-like morphology. Intercalation occurs quickly, within the first hour for the majority of cells. The rate-limiting step in the transmigration process appears to be disengagement of the UM cells from the EC monolayer and migration onto the underlying substrate. Integrin-based adhesion, especially involving VCAM, appears to play a role in maintaining adhesion of UM cells to ECs. BAP1 depletion appears to promote UM cell migration underneath the monolayer. This system may be an important new model for the study of metastasis in uveal melanoma.

## Supporting Information

S1 Movie **UM cells transmigrate through a novel intercalation process.** DIC movies of UM cells, 92.1 and OCM-1A, intercalating into the endothelial monolayer. Scale bar  = 50 µm. Frames were collected at a rate of one per 12 sec. The movies play at a rate of 30 frames per sec.
(AVI)

S2 Movie **Invasive projections are rich in F-actin.** UM cell (OCM-1A) with F-tractin-labeled actin filaments intercalating between ECs in a monolayer. F-actin-rich protrusions are seen transiently probe the space under the monolayer. The UM cell completes intercalation and maintains contact with adjacent ECs. Scale bar  = 20 µm. Frames were collected at a rate of one per 12 sec. The movies play at a rate of 15 frames per sec.
(AVI)

S3 Movie **Melanoma transmigration is a stepwise process.** Laser-scanning confocal movie of transmigration of a UM cell (OCM-1A) expressing F-tractin (pseudo-colored to show fluorescence intensity). Scale bar  = 25 µm. Frames were collected at a rate of one per 20 sec. The movies play at a rate of 30 frames per sec.
(AVI)
